# Intestinal protozoan infections among children 0-168 months with diarrhea in Mozambique: June 2014 - January 2018

**DOI:** 10.1371/journal.pntd.0008195

**Published:** 2020-04-22

**Authors:** Adilson Fernando Loforte Bauhofer, Idalécia Cossa-Moiane, Selma Marques, Esperança L. Guimarães, Benilde Munlela, Elda Anapakala, Jorfélia J. Chilaúle, Marta Cassocera, Jerónimo S. Langa, Assucênio Chissaque, Júlia Sambo, Lena Manhique-Coutinho, Diocreciano Matias Bero, Timothy A. Kellogg, Nilsa de Deus

**Affiliations:** 1 Direcção de Formação e Comunicação em Saúde, Instituto Nacional de Saúde (INS), Marracuene, Moçambique; 2 Instituto de Higiene e Medicina Tropical (IHMT), Universidade Nova de Lisboa, Lisboa, Portugal; 3 Direcção de Laboratórios de Saúde Pública, Instituto Nacional de Saúde (INS), Marracuene, Moçambique; 4 Institute of Tropical Medicine (ITM), Antwerp, Belgium; 5 Centro de Biotecnologia, Universidade Eduardo Mondlane, Maputo, Mozambique; 6 Direcção de Pesquisa em Saúde e Bem-Estar, Instituto Nacional de Saúde (INS), Marracuene, Moçambique; 7 Direcção de Inquéritos e Observação de Saúde, Instituto Nacional de Saúde (INS), Marracuene, Moçambique; 8 Institute for Global Health Sciences, University of California San Francisco, San Francisco, California, United States of America; Hitit University, Faculty of Medicine, TURKEY

## Abstract

**Background:**

Intestinal parasites such as *Cryptosporidium spp*., *Giardia lamblia* and *Entamoeba histolytica* can cause severe diarrhea, especially among children in developing countries. This study aims to determine the frequency of *Cryptosporidium spp*., *Giardia lamblia* and *Entamoeba histolytica* in children with diarrhea and identify risk factors for infection.

**Methodology:**

We conducted a cross-sectional study in children aged 0–168 months hospitalized with diarrhea in three regions of Mozambique, from June 2014 to January 2018. Following consent, caretakers were interviewed and a single stool specimen was collected from each child to diagnose *Cryptosporidium spp*., *G*. *lamblia* and *E*. *histolytica* using commercial immune-enzymatic assay (TechLab, Inc, Blacksburg, VA, USA). Anthropometric data were collected from the clinical reports. Multivariable logistic regression models were built to identify risk factors for *Cryptosporidium spp*. and *G*. *lamblia* infection.

**Results:**

Twenty-one percent of all specimens (212/1008) presented at least one parasitic infection. *Cryptosporidium spp*. infection was the most common 12.0% (118/985), followed by *G*. *lamblia* 9.7% (95/983) and *E*. *histolytica* 2.0% (20/1004). Risk factors for infection by *Cryptosporidium spp*. were: provenience (children from Nampula province showed the highest risk, OR: 8.176; CI: 1.916–34.894; p-value < 0.01); animal contact (children with animal contact had a protective effect OR: 0.627; CI: 0.398–0.986; p-value < 0.05); underweight (children severely underweight showed a risk of 2.309; CI: 1.310–4.069; p-value < 0.05). Risk factors for infection by *G*. *lamblia* were: age (group with highest risk, 60–168 months (OR: 2.322; CI: 1.000–5.393, p-value > 0.05)); and living in a household with five or more members (OR: 2.141; CI: 1.286–3.565, p-value < 0.01).

**Conclusions:**

Parasitic infection is common among children with diarrhea. Routine testing, standard treatment, and assessment for risk exposure of children with diarrhea should be implemented at health facilities in Mozambique.

## Author summary

Intestinal parasites can cause diarrhea in children, being most reported in developing countries, such as Mozambique. Mozambique dual high burden of HIV and malnutrition poses an ideal setting for parasites occurrence. To date, little information is published regarding intestinal parasites in children (0–168 months) in Mozambique, most of the published information focus on children under fifty-nine months and focus Mozambique’s South region. We determine *Cryptosporidium spp*., *Giardia lamblia* and *Entamoeba histolytica* frequency in the three regions of Mozambique in children hospitalized with diarrhea and we identified factors that are associated with intestinal parasite infections. *Cryptosporidium spp*. infection was the most common parasite among children in this study, with an occurrence of one in ten. The percentage of children infected with *Cryptosporidium spp*. decreased with age. *G*. *lamblia* was the second more common parasite, being the number of members in the household a predictor for its occurrence in children. *E*. *histolytica* was the less common of them all although it was found in all studied provinces. Our study highlights the burden of most common intestinal protozoans found in children (0–168 months) hospitalized due to diarrhea.

## Introduction

Globally, 6.3 million children (0–168 months) died in 2017, most of which were under five years old (5.4 million). Sub-Saharan Africa has the highest under-five mortality rate in the world, having one death in every 13 births [1]. In Mozambique, the under-five mortality is estimated to be between 50 to 70 deaths per 1000 births [1]. Diarrhea accounts globally for approximately 8% (432000) of all deaths in children less than five years old [1].

Infectious diarrhea can be caused by parasites, bacteria, viruses, or fungi [2]. Among intestinal parasites, *Cryptosporidium spp*. is most common in children with diarrhea, followed by *Giardia lamblia* and *Entamoeba histolytica* [2–6].

Having a developing immune system, being undernourished, and animal contact are risk factors for intestinal parasite infection [2,7,8]. Symptoms of intestinal parasites can include lack of appetite, anemia, abdominal pains, cognitive impairment, fever, vomiting, malabsorption, and diarrhea [2,7,8].

In Mozambique, previous studies have found an overall intestinal parasite prevalence ranging from 14.4% to 16.1% in children under five years [9,10]. Among protozoans, *G*. *lamblia* and *Cryptosporidium spp*. were more frequent and *E*. *histolytica/E*. *dispar* less common (18.6%, 17.7% and 10.2% respectively) [5]. Risk factors for parasitic infection include child age, poor nutrition status and HIV infection [4,5,11].

In a Global Enteric Multicenter Study (GEMS), including a site in Mozambique conducted in children less than five years old, *Cryptosporidium spp*. was considered the second most attributable pathogen to diarrhea, thus being essential to understand factors related to this agent [4].

All previously published studies were conducted in Mozambique’s south region and children younger than five years [4,5,9,10], although the occurrence of intestinal parasites in other regions of Mozambique in school-age children without diarrhea has been reported [12].

In 2014, a sentinel surveillance system was established to track diarrheal illness among children at selected hospitals in Mozambique. This study utilized data to assess enteric protozoan infections among children. The aims of this study are: 1) determine the frequency of *Cryptosporidum spp*., *G*. *lamblia* and *E*. *histolytica* in children aged 0–168 months admitted in the health facilities with diarrhea in three regions of Mozambique and 2) identify risk factors for parasitic infection.

## Methodology

### Study design

A cross-sectional study was conducted from June 2014 to January 2018. Data used in this analysis was obtained from the National Surveillance of Diarrhea (ViNaDia), an ongoing cross-sectional study of diarrhea illness among children in Mozambique. ViNaDia methods are described elsewhere [13], bellow is a description of study procedures for the surveillance of intestinal protozoan parasites only.

### Study sites

The ViNaDia system has been implemented in six health facilities in four provinces from the three regions of Mozambique: in the South region three Hospitals have been included (Hospital Central de Maputo, Hospital Geral de Mavalane and Hospital Geral José Macamo), the Central region includes two hospitals (Hospital Central da Beira and Hospital Geral de Quelimane) and the North region one hospital (Hospital Central de Nampula).

The surveillance initiated in Hospital Geral de Mavalane, and the other health facilities were gradually included. All study sites are located in urban areas.

The criteria for selecting health facilities were: 1) having a general pediatric outpatient clinic and an inpatient service; 2) ability to provide care to children living in adjacent neighborhoods and local districts except for Hospital Central de Maputo (a national reference hospital—none of the participants recruited in Hospital Central de Maputo were from center or north region of Mozambique); and 3) having a reliable specimen transport system to collect and store stool sample in a timely and appropriate manner.

### Study population, inclusion and exclusion criteria

The study population included children aged from 0 to 168 months who presented to the study health facility with diarrhea, defined as three or more loose liquid stools within the last 24 hours [14].

Children who acquired diarrheal disease during hospitalization or whose caretaker did not agree to sign the informed consent form were excluded from this study.

### Sample size

The expected minimum sample size was calculated using OpenEpi [15] with an assumed prevalence of 16.1% for intestinal parasites [9] based on previous studies. We obtained a minimum sample size of at least 208. However, in this study, we included all children who met the inclusion criteria during the study period.

### Data collection

We collected data using a structured data collection form. The data collection form was divided into sections: demographic information, clinical information, child immunization status and family socio economics status. Caretakers of eligible children were interviewed to collect additional information: sex, age, number of household members, clinical information and history of contact with animals (defined as having physical contact with any animal or their excrements in areas where the child circulated).

The weight of the children at the time of presentation was collected from the clinical records to assess the nutritional status. If the child’s weight was not indicated in the clinical records, the health professional weighed the child and recorded it on the data collection form. Children under two years were measured lying down, and children with two years or more were measured while they were standing. Data collection was conducted by trained health professionals assigned at each site. The study team provided all the materials used for data collection, and all data collectors were trained on study procedures.

### Sample collection and handling

One single stool specimen was collected from each study participant in a polystyrene tube and refrigerated in a cooler box with a temperature between 2ºC to 8ºC. Stool specimens collected in the Center and North regions were kept at -20ºC until shipment to the *Instituto Nacional de Saúde* (INS). Stool specimens collected in the South region were preserved in a cooler box and delivered to INS up to two hours after the collection.

Upon reception at the INS, samples were sent to three different laboratories (parasitology, microbiology and virology) and aliquots were made separately for virologic, parasitic, and bacterial diagnoses. The aliquot for parasitic diagnoses were stored at -40ºC freezer until laboratory testing.

### Laboratory detection of *Cryptosporidium spp*., *G*. *lamblia*, and *E*. *histolytica*

We used individual commercial immunoassays (TechLab, Inc, Blacksburg, VA, USA) to detect *Cryptosporidium spp*. oocysts antigen (CRYPTOSPORIDIUM *II*), *G*. *lamblia* cysts antigen (GIARDIA *II*) and *E*. *histolytica* cysts antigens (E. HISTOLYTICA *II*) according to the manufacturer recommendations [16–18]. *G*. *lamblia* and *Cryptosporidium spp*. were considered positive if the test results were ≥ 0.090 (absorbance at 450/620nm) and negative in test result < 0.090 (absorbance at 450/620nm) [16,18]. *E*. *histolytica* result was considered positive if the reading were 0.050 or higher after the negative control absorbance was subtracted, and was considered negative if the reading were < 0.050 after the negative control reading was subtracted [17].

### Data management

We double entered questionnaire data and laboratory results into an electronic database using Epi Info 3.5.1 (Centers for Disease Control and Prevention, Atlanta, 2008) and exported to Microsoft Excel 2016 for data cleaning. We determined each child’s nutritional status by calculating underweight (WAZ) Z-Scores using the WHO Anthro software version 3.2.2 (for children aged 0 to 59 months) and WHO Anthro Plus software version 1.0.4 (for children aged 60 to 168 months) [19,20]. Nutritional status was classified as: adequate for well-nourished (-2 ≤ z ≤ +2); and malnutrition was classified as moderate (-3 ≤ z < -2) and severe (-6 ≤ z < -3) according to the WHO standards [21]. Children overweight were excluded from nutritional status analysis (2 < z ≤ +5) [21]. Reference data used to calculate anthropometry was WHO.

### Statistical analysis

We analyzed the data using SPSS software (Statistical Package for the Social Sciences, Armok, NY: IBM Corp, 2011, version 25.0). We performed univariate analysis to describe the population characteristics and summarized the information in frequency tables. We performed a descriptive bivariate analysis for each parasite (*Cryptosporidium spp*., *G*. *lamblia*, and *E*. *histolytica*). All participants had complete data for the variables age and province. Two models were build using as dependent variables: *Cryptosporidium spp*. outcome and *G*. *lamblia* outcome. We performed crude bivariate logistic regression to identify factors associated with infection by *Cryptosporidium spp*. and *G*. *lamblia*. Independent variables that were associated with dependent variables at a level 0.1 in the crude bivariate logistic regression were included in the adjusted logistic regression model. Goodness-of-fit was based on Hosmer and Lemeshow test. Variables that showed a p-value of less than 0.05 were considered statistically significant.

### Ethical statement

The National Bioethical Committee for Health from Mozambique approved the protocol (IRB00002657, reference Nr: 348/CNBS/13). Caretakers of eligible children provided written informed consent after receiving information about the purpose of the study. We ensured the confidentiality of all participants by storing the physical data collection and consent forms in a lockable cabinet with access only to the study investigators. In addition, the computers with study stored data were accessible by investigators with an access code.

## Results

During the study period, 1978 children with diarrhea who presented at one of the study health facilities were enrolled in the survey of which 85.5% (1751/1978) provided a stool sample for testing. Of the specimens collected, 4.0% (70/1751) were lost during the transport process to INS, less than 1.0% (4/1681) were not tested due to incomplete completion of the laboratory paperwork and 39.8% (669/1681) samples were insufficient for testing at least one parasite. In total, the laboratory tested 1008 specimens (60.0%).

### Population characteristics

The majority of children in the sample were male (59.2%). Most of the children were: aged from 0–11 months (48.7%), were enrolled from the health facilities in Maputo province (53.9%), lived in a household with five or more members (59.1%), and drinking water came from a piped water source (56.1%) (Table 1). Overall, 45.1% of the children had animal contact, and 4.9% were HIV positive (Table 1).

**Table 1:** Characteristics of children hospitalized with diarrhea.

Characteristics	N = 1008	%
***Sex***		
Male	597	59.2
Female	410	40.7
Unknown/ missing	1	0.1
		
***Age group (months)***		
0–11	491	48.7
12–23	339	33.6
24–59	123	5.5
60–168	55	12.2
		
***Province***		
Maputo	543	53.9
Sofala	52	5.2
Zambézia	128	12.7
Nampula	285	28.3
		
***Household members***		
<5	373	37.0
≥5	596	59.1
Unknown/ missing	39	3.9
		
***Animal contact***		
Yes	455	45.1
No	523	51.9
Unknown/ missing	30	3.0
		
***Drinking Water Source***		
Public tap water	320	31.7
Piped water	565	56.1
Tube well	95	9.4
Others	14	1.4
Unknown/ missing	14	1.4
		
***HIV Status***		
Positive	49	4.9
Negative	437	43.4
Unknown/ missing	522	51.8
		
***Nutritional Status***		
*Underweight*		
Adequate	577	57.8
Moderate	164	16.4
Severe	132	13.2
Unknown/ missing	135	12.5

N: Total number of samples tested

### Overall frequency of intestinal protozoans (*Cryptosporidium spp*., *G*. *lamblia* and *E*. *histolytica*)

**Fig 1:**
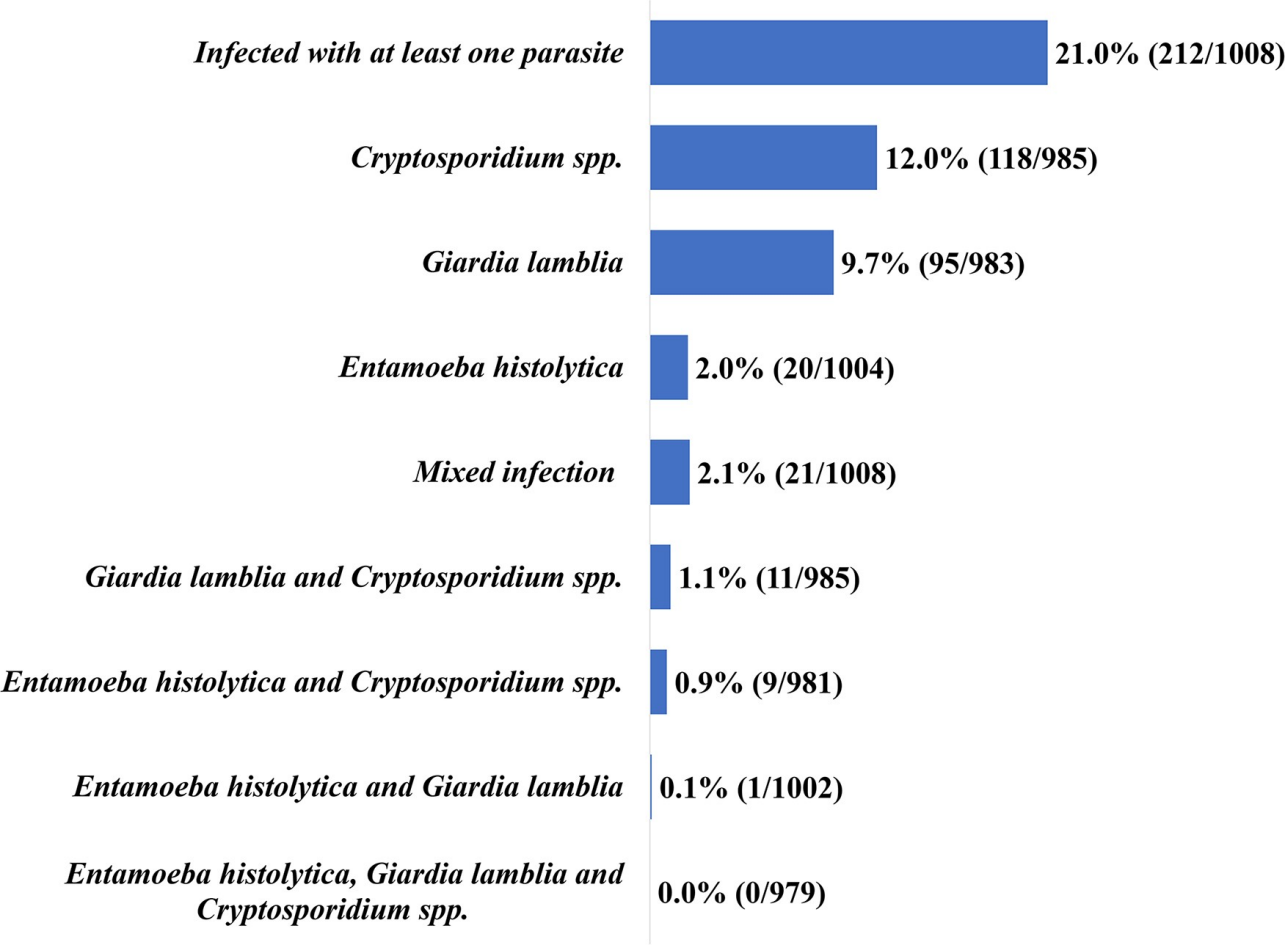
Frequency of intestinal protozoans (*Cryptosporidium spp*., *G*. *lamblia* and *E*. *histolytica*).

The overall percentage of at least one intestinal parasite was 21.0% (212/1008), with *Cryptosporidium spp*. being the most common infection at 12.0% (118/985), followed by *G*. *lamblia* at 9.7% (95/983) and *E*. *histolytica* the least common at 2.0% (20/1004). Co-infection was observed in 2.1% (21/1008) of children with the combination of *G*. *lamblia* and *Cryptosporidium spp*. the most common at 1.1% (11/985) (Fig 1).

### Frequency and Risk Factors for *Cryptosporidium spp*. infection

We analyzed data from 985 cases, and we found 118 positive cases (12.0%) for *Cryptosporidium spp*. Three of the four provinces (Maputo, Sofala and Nampula) showed a similar frequency for *Cryptosporidium spp*. infection (13.0%), except for Zambézia (4.0%). In multivariable analysis, infection with *Cryptosporidium spp*. was more likely for children living in Nampula province (adjusted odds ratio: 8.176, 95% CI: 1.916–34.894, p-value < 0.01) (Table 2).

**Table 2:** Descriptive characteristics, bivariate and multivariate analysis of children infected by *Cryptosporidium spp*.

Characteristics	N = 985	n = 118	% (Positive)	Bivariate OR (95% CI)	Adjusted OR (95% CI)
***Sex***					
Male	581	66	11.4	Ref	NA
Female	403	52	12.9	1.156 (0.784–1.704)	NA
Unknown/ missing	1	0	0		
***Age group (months)***					
0–11	478	64	13.4	2.680 (0.813–8.836)	NA
12–23	331	42	12.7	2.519 (0.753–8.430)	NA
24–59	121	9	7.4	1.393 (0.362–5.359)	NA
60–168	55	3	5.5	Ref	NA
***Province***					
Maputo	525	68	13.0	3.601 (1.421–9.127)**	6.054 (1.433–25.583)*
Sofala	51	6	11.8	3.227 (0.938–11.096)	5.839 (1.112–30.653)*
Zambézia	126	5	4.0	Ref	Ref
Nampula	283	39	13.8	3.868 (1.487–10.064)**	8.176 (1.916–34.894)**
***Household members***					
<5	366	44	12.0	1.012 (0.676–1.514)	NA
≥5	580	69	11.9	Ref	NA
Unknown/ missing	39				
***Animal contact***					
Yes	448	43	9.6	0.641 (0.429–0.958)*	0.627 (0.398–0.986)*
No	507	72	14.2	Ref	Ref
Unknown/ missing	30				
***Drinking Water Source***					
Public tap water	315	39	12.4	1.159 (0.554–2.422	NA
Piped water	550	63	11.5	1.061 (0.523–2.151)	NA
Tube well	92	10	10.9	Ref	NA
Others	14	2	14.3	1.367 (0.267–7.007	NA
Unknown/ missing	14				
***HIV Status***					
Positive	49	7	14.3	1.088 (0.466–2.538)	NA
Negative	429	57	13.3	Ref	NA
Unknown/ missing	507				
***Nutritional Status***					
*Underweight*					
Adequate	560	48	8.6	Ref	Ref
Moderate	160	29	18.1	2.361 (1.433–3.891)**	2.552 (1.521–4.283)***
Severe	130	21	16.2	2.055 (1.182–3.572)*	2.309 (1.310–4.069)**
Unknown/ missing	135				

N: Total number of samples tested; n: number of positive samples; NA: not applicable

*: P < 0.05

**: P < 0.01

***: P < 0.001

Children with reported animal contact were significantly less likely to be infected by *Cryptosporidium spp*. compared with the children without reported animal contact (9.6%, 43/448 vs 14.2%, 72/507 respectively, adjusted odds ratio: 0.627; CI: 0.398–0.986, p-value < 0.05 (Table 2).

Children showing a moderate or severe status for underweight showed a higher risk of infection by *Cryptosporidium spp*. compared with the children who were well-nourished adjusted odds ratio: moderate (adjusted OR: 2.552; CI: 1.521–4.283, p-value < 0.001), severe (aOR: 2.309; CI: 1.310–4.069, p-value < 0.01) (Table 2).

In this sample, sex, age, number of household members, animal contact, drinking water source, and HIV status were not associated with *Cryptosporidium spp*. infection among children with diarrhea (Table 2).

### Frequency and Risk Factors for *G*. *lamblia* infection

We analyzed data from 983 cases, and we found 95 children infected (9.7%) with *G*. *lamblia*. Children age 12–23 months were more likely to be infected by *G*. *lamblia* (12.1%; 40/330) compared to children age 0–11 months (6.5%; 31/477), adjusted odds ratio: 1.870 (CI: 1.137–3.075, p-value < 0.05 (Table 3).

**Table 3:** Descriptive characteristics, bivariate and multivariate analysis of children infected by *G*. *lamblia*.

Characteristics	N = 983	n = 95	% (Positive)	Bivariate OR (95% CI)	Adjusted OR (95% CI)
***Sex***					
Male	580	61	10.5	1.272 (0.819–1.976)	NA
Female	402	34	8.5	Ref	NA
Unknown/ missing	1				
***Age group (months)***					
0–11	477	31	6.5	Ref	Ref
12–23	330	40	12.1	1.984 (1.214–3.245)**	1.870 (1.137–3.075)*
24–59	121	14	11.6	1.882 (0.968–3.662)	1.759 (0.868–3.563)
60–168	55	10	18.2	3.197 (1.472–6.946)**	2.322 (1.000–5.393)
***Province***					
Maputo	523	52	9.9	Ref	NA
Sofala	51	0	0.0	NA	NA
Zambézia	126	14	11.1	1.132 (0.606–2.115)	NA
Nampula	283	29	10.2	1.034 (0.640–1.670)	NA
***Household members***					
<5	364	21	5.8	Ref	Ref
≥5	580	69	11.9	2.205 (1.138–3.663)**	2.141 (1.286–3.565)**
Unknown/ missing	39				
***Animal contact***					
Yes	447	48	10.7	1.232 (0.803–1.891)	NA
No	506	45	8.9	Ref	NA
Unknown/ missing	30				
***Drinking Water Source***					
Public tap water	315	29	9.2	1.318 (0.166–10.441)	NA
Piped water	548	53	9.7	1.392 (0.179–10.851)	NA
Tube well	92	8	8.7	1.238 (0.143–10.729)	NA
Others	14	1	7.4	Ref	NA
Unknown/ missing	14				
***HIV Status***					
Positive	49	2	4.1	Ref	NA
Negative	429	43	10.0	2.618 (0.614–11.157)	NA
Unknown/ missing	505				
***Nutritional Status***					
*Underweight*					
Adequate	559	51	9.1	Ref	NA
Moderate	159	13	8.2	0.887 (0.469–1.676)	NA
Severe	130	14	10.8	1.202 (0.644–2.246)	NA
Unknown/ missing	115				

N: Total number of samples tested; n: number of positive samples; NA: not applicable

*: P < 0.05

**: P < 0.01

*G*. *lamblia* infection was more common in children living in a household with five or more members (11.9%; 69/580) when compared with the ones living in a household with less than five household members (5.8%; 21/364), adjusted odds ratio: 2.141 (CI: 1.286–3.565, p-value < 0.01) (Table 3).

### Frequency and Risk Factors for *E*. *histolytica* infection

We analyzed data from 1004 cases, and we found 20 positive cases (2.0%). In males *E*. *histolytica* was more common 2.5% (15/595). Children age 24–59 months were most affected with *E*. *histolytica* (3.3%; 4/123) (Table 4).

**Table 4:** Descriptive characteristics of children infected by *E*. *histolytica*.

Characteristics	N = 1004	n = 20	% Positive
***Sex***			
Male	595	15	2.5
Female	408	5	1.2
Unknown/ missing	1	0	0.0
			
***Age group (months)***			
0–11	489	9	1.8
12–23	337	6	1.8
24–59	123	4	3.3
60–168	55	1	1.8
			
***Province***			
Maputo	541	17	3.1
Sofala	51	1	2.0
Zambézia	128	1	0.8
Nampula	284	1	0.4
			
***Household members***			
<5	372	7	1.9
≥5	593	13	2.2
Unknown/ missing	39	0	0.0
			
***Animal contact***			
Yes	454	7	1.5
No	520	13	2.5
Unknown/ missing	30	0	0.0
			
***Drinking Water Source***			
Public tap water	319	4	1.3
Piped water	563	14	2.5
Tube well	94	1	1.1
Others	14	0	0.0
Unknown/ missing	14	1	7.1
			
***HIV Status***			
Positive	49	2	4.1
Negative	436	9	2.1
Unknown/ missing	519	9	1.7
			
***Nutritional Status***			
*Underweight*			
Adequate	576	5	0.9
Moderate	164	4	2.4
Severe	130	6	4.6
Unknown/ missing	114	5	4.4

N: Total number of samples tested; n: number of positive samples

*E*. *histolytica* infection frequency varied by provinces, Maputo showed the highest frequency (3.1%; 17/541) and Nampula had the lowest (0.4%; 1/284). *E*. *histolytica* frequency among households with five or more members was similar to households with fewer members (2.2% versus 1.9%) (Table 4).

Bivariate and multivariate analysis was not performed for *E*. *histolytica* due to the low number of positives. Alternatives approach was taken, by using the Chi-square test with continuity correction or Fisher’s exact test, in all cases no association was observed between the presence of *E*. *histolytica* with the independent variables.

## Discussion

In this cross-sectional study, we found that parasitic infections were common, one-in-five children with diarrhea were infected with one or more intestinal parasites, presenting a serious public health problem. The above finding from this study is higher than previous studies conducted in Mozambique’s South region with an intestinal parasite’s frequency of 14.4% and 16.1% [9,10]. Differences observed from the earlier studies may be due to the microscopic diagnostic technique used in those studies, which is less sensitive than the immune enzymatic technique we used in this study [22], or we may consider an increase of parasitic infection due to inclusion of multiple sites in different regions.

Our finding showed that *Cryptosporidium spp*. was the most common intestinal parasite found among children with diarrhea which is consistent with the Global Enteric Multicenter Study, conducted between December 2007 to March 2011 which reported *Cryptosporidium spp*. as the primary protozoan attributed as the cause of diarrhea in children [4,5].

*G*. *lamblia* was the second most common parasite and *E*. *histolytica* was the less common. For both, the proportion was lower when compared to a previous study conducted in a rural setting in the south region of Mozambique (18.8% for *G*. *lamblia* and 10.2% for *E*. *histolytica/E*. *dispar*) using the same diagnostic technique [5]. Previous studies reported a high frequency of intestinal parasites in rural settings compared to urban settings [23,24]. This finding maybe because of inadequate water supply, improper drainage systems and difficulty in accessing drug pharmacies to obtaining treatment against enteric parasites in rural settings [24].

The surveillance of *E*. *histolytica* is being carried out in many countries, reporting higher frequencies than the one we found in Mozambique, most of them primarily using microscopic techniques which cannot distinguish between pathogenic and non-pathogenic (*Entamoeba dispar* and *Entamoeba moshkovskii*), leading to over estimation of the true occurrence of *E*. *histolytica* [25–27]. In Mozambique, few studies reported *E*. *histolytica* using microscopy, only one of them included a molecular diagnostic approach and was unable to detect *E*. *histolytica*, only found *E*. *dispar* [10,28]. The study conducted in Manhiça—south region of Mozambique that used an ELISA technique to report *E*. *histolytica* infections, used an ELISA technique which reported a non-conclusive result, it reported infections by *E*. *histolytica/E*. *dispar*, our results may differ from this study because we used an ELISA kit specific only for *E*. *histolytica* [5]. Probably most of the positive children for *E*. *histolytica/E*. *dispar* were infected only by *E*. *dispar*, to confirm this hypothesis, positive samples previously reported as positive should be tested by a specific ELISA kit for *E*. *histolytica* such as the one we used or by a molecular diagnose tool for *E*. *histolytica*.

A case-control study done in Mozambique reported a similar proportion of *E*. *histolytica/E*. *dispar* in case and controls regardless of their age, using an ELISA technique, which raises the question of whether *E*. *histolytica/ E*. *dispar* alone can generate symptomatology (e.g. diarrhea) [5]. Others may say *E*. *histolytica* is only found in symptomatic patients if there is co-infection with another enteric pathogen able to cause diarrhea [26]. We exclusively focused on intestinal protozoan detection and we found co-infection between: *E*. *histolytica* and *G*. *lamblia*, *E*. *histolytica* and *Cryptosporidium spp*. It is likely that if our surveillance system tested for other pathogens, other co-infections with *E*. *histolytica* would have been detected [26].

*Cryptosporidium* infection was observed in all study sites. This finding highlights the importance of studying this parasite in other Mozambique health facilities not included in the present study, to better estimate parasites distribution among children with diarrhea in the country. Zambézia province, showed less frequency, compared to the other provinces, suggesting different geographic distribution of *Cryptosporidium spp*. in Mozambique. Parasite geographic distribution can vary as it was previously observed in a country wide survey on enteric parasites in schoolchildren [12]. However, further studies should be conducted to better understand the factors related to host, environment and behavior which may explain the low frequency of *Cryptosporidium spp*. in Zambézia province. Additionally, knowing which *Cryptosporidium* species circulates in each site may provide evidence of the pathway used in transmission and by that, easy to see which barriers exist in Zambézia that reduce the frequency of *Cryptosporidium spp*.

Although contact with animals has been reported to be a risk factor for *Cryptosporidium spp*. infection, we observed a higher frequency of the pathogen in children without animal contact. This finding suggests that the transmission of *Cryptosporidium spp*. oocysts may be by person-to-person contact, which has been more recorded in Africa [2,29]. More research is needed, using molecular diagnosis tools, to understand *Cryptosporidium spp*. transmission pathways [29].

Mozambique is one of the countries most affected by HIV with a prevalence of 13.2% [30]. *Cryptosporidium spp*. infection is expected to be higher in patients with HIV infection compared to patients without HIV infection. HIV positive children become progressively immunosuppressed, increasing the chance of acquiring infections [11]. In the GEMS study, *Cryptosporidium spp*. was considered a significant pathogen regardless of HIV infection [4], in our study we saw similar frequency of *Cryptosporidium spp*. in children HIV positive and negative. Similar frequency of *Cryptosporidium spp*. among HIV and non-HIV children can be due to the immune reconstitution with anti-retroviral therapy among HIV positive children [31]. The other reason can be the fact that *Cryptosporidium spp*. is more frequent in children HIV positive, with persistent and chronic diarrhea, and our study included children with acute diarrhea [11].

Mozambique is one of the most affected countries by malnutrition (43.0% for stunting, 15.0% for underweight and 6.0% for wasting) according to health demographic survey [32]. Underweight was related to *Cryptosporidium spp*. infection. Parasitic infection and malnutrition have been recorded with similar geographic distribution, not knowing if *Cryptosporidium spp*. infection caused malnutrition or if malnourishment makes children more vulnerable to infection [29]. Parasitic infection is thought to lead to child nutritional loss, chronic inflammation and subtle reduction in digestion and absorption. Moreover, being malnourished would mean that the immune system would not adequately protect the child from an opportunistic infection—by *Cryptosporidium spp*. [33–35]. Unfortunately, our cross-sectional study design does not allow us to answer the bi-directional relation between *Cryptosporidium spp*. and malnutrition.

We observed that *G*. *lamblia* infection was more common in older children. This finding was also observed in other studies; -in hospitalized children from Manhiça district in the south region of Mozambique, Brazil and Cuba [5,7,36,37]. Children’s habits can explain this finding. As children grow they have more contact with environments which can be contaminated by *G*. *lamblia*, while younger children with less mobility have less exposure, and have additional protection from breastfeeding [9]. Interestingly the opposite was observed for *Cryptosporidium spp*. being more common in younger children and less common in the older ones, as for *E*. *histolytica* distribution were similar across the ages, raising a real concern in factors related to behavior and host that may predispose infection (no statistical support was observed between age and infection by *Cryptosporidium spp*. or *E*. *histolytica*).

*G*. *lamblia* infection was more common in children living with five or more members in their houses. This finding was also reported in children hospitalized with diarrhea in Brazil where number of children in the household was a risk for infection, suggesting that the type of transmission is person-to-person contact in crowed environments [36].

All three studied parasites are known to be waterborne, yet, no association with drinking water source was found, in many cases when parasites are related to water are in known outbreaks, which was not recorded in the study population during the recruitment period [29]. Regardless of the water source, boiling or filtration can prevent infection [22].

We could not find any risk factors related to *E*. *histolytica*, which may indicate sanitation as the main determinant for acquiring infection by *E*. *histolytica* [26]. We found a similar proportion of *E*. *histolytica* in children HIV positive with the one reported in Tanzania, which is Mozambique neighbor country in the same study a high relation between *E*. *moshkovskii* and HIV positive was observed [38].

Although no statistical difference was observed between provinces for *E*. *histolytica*, the provinces near Tanzania—where *E*. *histolytica* was not detected in a case-control study in children younger than 2 years—(Nampula and Zambézia), we observed in our study lower frequencies than Sofala and Maputo provinces, suggesting that geographic distribution may have a role in *E*. *histolytica* occurrence [6].

We identify three main limitations of our study. First, we only tested one stool sample for each participant, some studies recommend testing multiple samples for each participant to avoid underestimation of the true frequency [9,22,39], although we were not able to increase the number of stool specimen collected for each participant, we used a more sensitive diagnose technique [40]. We had many missing information in the independent variables; however, our sample size meets the minimal sample size required for inferential analysis. Our laboratory procedures were unable to diagnose helminth infections in recruited children.

### Conclusions

Parasitic infection is common among children with diarrhea. *Cryptosporidium spp*. was the most common parasite in Maputo, Sofala and Nampula provinces. There are more factors related to *Cryptosporidium spp*. infection to be explored across the provinces, using a different study design to better understand its relation with malnutrition. *Cryptosporidium spp*. was more common in younger children than the older ones, the opposite was observed for *G*. *lamblia*. A large number of household members in the child’s household and child age were factors related to infection by *G*. *lamblia* infection. *E*. *histolytica* was found in all three regions of the country although in a lower frequency.

### Recommendations

Routine testing, standard treatment, and assessment for risk exposure should be implemented at health facilities in Mozambique for children admitted with diarrheal disease. Additional studies using different designs such as longitudinal approach should be done to better explore type of relation between *Cryptosporidium spp*. and malnutrition. *Cryptosporidium spp*. molecular characterization would provide information regarding which species circulate in Mozambique and transmission pathways, either by animal contact or person-to-person contact. Anti-retroviral therapy role against *Cryptosporidium spp*. infection should be better understood. Other pathogens associated with *E*. *histolytica* such as virus and bacteria should be taken in count in future studies.

## Supporting information

S1 Checklist STROBE checklist. (DOCX)

S1 Data (XLSX)
